# Vagus Nerve Stimulation Enhances Extinction of Conditioned Fear in Rats and Modulates Arc Protein, CaMKII, and GluN2B-Containing NMDA Receptors in the Basolateral Amygdala

**DOI:** 10.1155/2016/4273280

**Published:** 2016-11-09

**Authors:** Amanda C. Alvarez-Dieppa, Kimberly Griffin, Sheridan Cavalier, Christa K. McIntyre

**Affiliations:** University of Texas at Dallas, 800 W Campbell Rd., Richardson, TX 75080, USA

## Abstract

Vagus nerve stimulation (VNS) enhances the consolidation of extinction of conditioned fear. High frequency stimulation of the infralimbic cortex (IL) produces long-term potentiation in the basolateral amygdala (BLA) in rats given VNS-paired extinction training, whereas the same stimulation produces long-term depression in sham-treated rats. The present study investigated the state of synaptic plasticity-associated proteins in the BLA that could be responsible for this shift. Male Sprague-Dawley rats were separated into 4 groups: auditory fear conditioning only (fear-conditioned); fear conditioning + 20 extinction trials (extended-extinction); fear conditioning + 4 extinction trials paired with sham stimulation (sham-extinction); fear conditioning + 4 extinction trials paired with VNS (VNS-extinction). Freezing was significantly reduced in extended-extinction and VNS-extinction rats. Western blots were used to quantify expression and phosphorylation state of synaptic plasticity-associated proteins such as Arc, CaMKII, ERK, PKA, and AMPA and NMDA receptors. Results show significant increases in GluN2B expression and phosphorylated CaMKII in BLA samples from VNS- and extended-extinction rats. Arc expression was significantly reduced in VNS-extinction rats compared to all groups. Administration of the GluN2B antagonist ifenprodil immediately after fear extinction training blocked consolidation of extinction learning. Results indicate a role for BLA CaMKII-induced GluN2B expression and reduced Arc protein in VNS-enhanced extinction.

## 1. Introduction

Extinction of conditioned fear is the process by which repeated exposure to a cue in the absence of the reenforcer leads to reduced expression of the conditioned fear response. This process requires the formation of a new memory that competes with the fear memory, thereby diminishing the fear response [1]. The persistence of strong aversive memories and the inability to extinguish fear are at the basis of disorders like phobias, panic disorder, obsessive compulsive disorder, and posttraumatic stress disorder [2–4]. Interventions that enhance extinction of fear could serve as treatments for disorders that present pathological fear and anxiety.

Vagus nerve stimulation (VNS) is a therapy approved by the FDA for the treatment of epilepsy and drug-resistant depression. Research shows that when administered immediately after training, VNS enhances memory consolidation in humans and in rats [5, 6]. Administration of VNS to rats during trials of fear extinction reduces expression of conditioned fear faster than extinction training alone [7], suggesting that VNS could be an effective adjunct to exposure therapies used to treat trauma-related and anxiety disorders. However, the mechanisms by which VNS can modulate and enhance consolidation of fear extinction are not known. Plasticity in the pathway between the infralimbic region of the prefrontal cortex (IL) and the basolateral complex of the amygdala (BLA) is implicated in extinction learning [1, 8]. For instance, a study suggests that the excitatory synaptic strength in the medial prefrontal cortex- (mPFC-) BLA pathway decreases after extinction of fear [8]. We recently reported that high frequency stimulation administered to the IL produced long-term depression (LTD) in field potentials in the BLA in fear conditioned, but not naive rats. Fear-conditioned animals given sham stimulation during extinction training also demonstrated LTD in this pathway; however, the same high frequency stimulation of the IL produced long-term potentiation (LTP) in the BLA in VNS-treated rats [9]. Extensive extinction training without VNS reversed the fear conditioning-induced LTD response to IL stimulation, possibly through LTP-like mechanisms, but did not result in LTP. A reduction in excitatory synaptic strength in the mPFC-BLA pathway [8] could allow for potentiation to more easily occur in the BLA when high frequency stimulation is applied to the IL [9]. These findings raise questions about the molecular mechanisms by which VNS enhances extinction of fear and influences plasticity in the IL-BLA pathway.

The calcium/calmodulin-dependent protein kinase (CaMKII) is a synaptic protein that undergoes autophosphorylation at Thr286 when activated and plays an important role in induction and maintenance of LTP by interacting with both AMPA and NMDA receptors [10]. Active CaMKII affects AMPA receptors during the early stages of LTP through phosphorylation of the GluA1 subunit of the AMPA receptor protein at S831, which in turn increases AMPA receptor conductance [11–13]. On the other hand, dephosphorylation of GluA1 at Ser845, which is targeted by cyclic-AMP protein kinase (PKA), is associated with LTD [13]. Exocytosis of AMPA receptors into the synaptic membrane, a mechanism thought to be important for later stages of LTP, depends in part on the RAS-extracellular regulated kinase (ERK) pathway, a pathway that can be stimulated by CaMKII [14, 15]. Activated CaMKII diffuses to the synapse and binds to the GluN2B subunit of NMDA receptors, a process that plays a role in LTP induction [16, 17]. Research on neuronal cell-cultures shows that these CaMKII/NMDA receptor complexes are persistent, suggesting that they might be involved in LTP maintenance as well [18]. Furthermore, studies suggest that GluN2B containing NMDA receptors in the BLA plays a role in amygdala-dependent conditioning [19, 20] and extinction learning [21].

Immediate early genes (IEGs) are studied as a measure of experience/activity related synaptic plasticity. The activity-regulated cytoskeletal-associated protein (Arc) is a commonly studied IEG because it plays a critical role in memory-related plasticity [22]. For example, intra-BLA infusions of a memory enhancing beta-adrenergic agonist immediately after an inhibitory avoidance (IA) task leads to increased Arc expression in the hippocampus, whereas pretraining intrahippocampal infusions of an antisense oligodeoxynucleotide, which prevents Arc translation, leads to impaired long-term memory for this task [23]. Furthermore, research shows that Arc expression in the amygdala is required for consolidation of auditory fear conditioning [24]. Studies also show involvement of Arc in synaptic plasticity. Evidence provided by* in vitro *electrophysiology studies suggest that Arc plays a role in LTD by regulating AMPA receptor cycling [25, 26].

Experiments in the present study were designed to test the hypothesis that pairing VNS with extinction training predisposes synapses to induction of LTP by shifting the balance of synaptic plasticity-associated proteins like Arc and CaMKII and, in turn, affecting receptor activity. To test this hypothesis, rats were subjected to auditory fear conditioning followed by fear extinction training in the presence or absence of VNS. After completion of the behavioral training, immunoblotting techniques were used to analyze differences in the balance of plasticity-related proteins in the BLA. Levels and phosphorylation states of CaMKII and downstream targets known to be involved in synaptic plasticity like ERK, Arc, the GluA1 subunit of AMPA receptors, and the GluN2A and GluN2B subunits of NMDA receptors were measured in the BLA after auditory fear conditioning or after either extinction training paired with VNS or sham stimulation or extended-extinction training without stimulation.

## 2. Materials and Methods

### 2.1. Animals

Male Sprague-Dawley rats (Charles River Breeding Laboratories, Wilmington, MA), 2-3 months old, were housed in a vivarium room, were kept on a 12-hour light/dark cycle, and had* ad libitum* access to food and water. All animal procedures were conducted in accordance with the University of Texas at Dallas Institutional Animal Care and Use Committee.

### 2.2. Surgery

The VNS surgeries were performed as previously described [27, 28]. Briefly, platinum-iridium wire electrodes were housed in custom-made Micro-Renathane cuffs (0.04 in. i.d., 0.08 in. o.d., and 4 mm long). Rats were anesthetized with isoflurane (2% in O_2_; Western Medical Supply) and positioned in a stereotaxic frame. A head implant was constructed using bone screws and dental acrylic to position and fix the platinum-iridium wires to the top of the skull. The vagus nerve was accessed through a small cervical incision on the left ventral side. The muscles were separated and the vagus nerve and carotid artery were exposed. The vagus nerve was isolated and the electrode was wrapped around the nerve. The left vagus nerve was targeted; the right vagus nerve innervates the sinoatrial node and can cause cardiac arrest [29]. Brief cessation of breath was observed following VNS (0.2 mA, 60 Hz, and 10 sec) delivery to anesthetized rats, confirming stimulation of vagal fibers. Sham-stimulated rats underwent the same surgical procedure; however, the circuit was designed to be short at the level of the head implant (i.e., after implantation of the headcap, the vagus nerve was exposed and isolated but no electrode was placed around the nerve). Rats were allowed 7 days to recover from surgery. All rats were handled 5 min/day for 5 days before behavioral experiments.

### 2.3. Apparatus

Rats underwent auditory fear conditioning and fear extinction training in 1 of 2 identical operant boxes built of Plexiglas walls (20 × 20 × 20 cm), with stainless steel rod flooring that was connected to a shock generator. The operant boxes were each located inside a sound-insulated chamber. A house light lit the chamber, while a digital camera located above the operant box recorded behavioral procedures. The session was viewed and monitored on a computer located outside the behavior room. Videos were saved for later analysis.

### 2.4. Auditory Fear Conditioning (AFC)

On day 1, rats were placed in a sound-insulated chamber where they were presented with 5 tones before conditioning in order to establish that the rats were not innately afraid of tone presentations. This was followed by 8 tone presentations (9 kHz, 75 dB SPL, and 30 s duration) that coincided with 0.5 mA footshock lasting 1 s. Twenty-four hours later, rats underwent a second round of auditory fear conditioning identical to that of day 1. Each tone stimulus was presented at an interval of 4 min, on average (3–5 min range), and the 1 s footshock occurred at a random time during the 30-second tone. The chamber was cleaned with 20% ethanol between subjects.

### 2.5. Conditioned Fear Response Test (CFRT)

Twenty-four hours after auditory fear conditioning (day 3), 4 tones were presented in the absence of footshock. The session was recorded by a digital camera, and time spent freezing during tone exposures was measured by investigators who were blind to the treatment group. Percentage of time spent freezing served as a measure of the conditioned fear response. Freezing was defined as complete immobility and posture consisting of lowered head, spread paws, and rapid respiration [30].

### 2.6. Extinction Training (EXT)

On day 4, rats were presented with 4 tones in the absence of footshock. During extinction trials, tones were paired with either VNS or sham stimulation. The vagus nerve or sham stimulation was delivered 150 ms before the onset of the tone, at an intensity of 0.4 mA, 500 *μ*s pulse width at 20 Hz, and for duration of 30 s.

### 2.7. Extended Extinction (EE)

Rats underwent AFC on days 1 and 2, followed by a CFRT on day 3. On day 4, during fear extinction training, 1 group of rats that did not undergo operation received 20 tones presentations in the absence of footshock or stimulation.

### 2.8. Tissue Preparation

Rats were euthanized 45 min after completion of extinction training. Animals were anesthetized using isoflurane (Western Medical Supply), and brains were rapidly removed and flash-frozen in cold 2-methylbutane. Brains were kept at −80°C for later molecular analysis. Coronal cryosection procedures of 500 *μ*m of thickness were taken, and the BLA was dissected using a tissue punch kit 0.5–0.9 mm in diameter. The tissue was then stored at −80°C to be used for immunochemical analysis.

### 2.9. Western Blots

The tissue was suspended in lysis buffer (0.1 M phosphate buffer, pH 7.4, 10% glycerol, 10% phosphatase inhibitor, and 20% protease inhibitor) and sonicated. Protein amounts were assessed using a Qubit protein assay kit and fluorometer (Life Technologies). Equal amounts of protein (15 *μ*g) for each sample were loaded into 4–12% gradient Bis-Tris MIDI gels (Life Technologies) and separated by electrophoresis. Proteins were then transferred from the gel onto a nitrocellulose membrane by electroblotting, using an iBlot dry-blotting system (Life Technologies). The membranes were blocked with 5% milk for one hour at room temperature. The membranes were immunoblotted with antibodies against Arc (rabbit; 1 : 1000; Synaptic Systems), p-CaMKII and CaMKII*α* and CaMKII*β* (rabbit; 1 : 500; Cell Signaling), p-PKA and PKA (rabbit; 1 : 1000; Cell Signaling), p-ERK and ERK (rabbit; 1 : 1000; Cell Signaling), p-GluA1 at Ser831 and p-GluA1 at Ser845 (rabbit; 1 : 500; Millipore), GluA1 (rabbit; 1 : 500; Cell Signaling), GluN2A and GluN2B (rabbit; 1 : 500; Millipore), and PSD95 (rabbit; 1 : 1000; Cell Signaling) and incubated overnight at 4°C. Membranes were then probed with secondary HRP-linked antibody (goat anti-rabbit; Cell Signaling) and incubated for 1 h at room temperature. A tris-buffered saline (TBS: 150 mM NaCl, 100 mM tris base, pH 7.5) solution containing 0.5% Tween was used to wash the membrane after incubation. Detection of immunoreactivity was determined by chemiluminescence (ECL Western Blot Kit, Pierce). Immunoblotting was normalized by comparison of the amount of protein of interest to total amount of PSD95 or actin protein loaded in the same sample. Tissue from all groups was loaded into adjacent wells within a gel for fair comparison. After imaging, films were scanned and analyzed using Image-J analysis software (NIH).

### 2.10. Data Analysis

For protein analysis, differences across groups were analyzed using an unpaired *t*-test or ANOVA with Fisher's Protected LSD* post hoc* test. For behavioral analysis, two-way ANOVA with repeated measures and Dunnett's multiple comparisons test was used. A probability level of less than 0.05 was considered statistically significant.

### 2.11. Drug Preparation and Administration

Ifenprodil tartrate salt (Sigma Aldrich), a noncompetitive, selective antagonist for GluN2B containing NMDA receptors, was dissolved in distilled water for systemic administration. To determine the influence of GluN2B receptor blockade on VNS modulation of consolidation of extinction, immediately after extinction training or extended extinction, rats were injected with 5 mg/kg of ifenprodil or vehicle (i.p.) and returned to their cages. On day 5 rats were subjected to a CFRT drug-free.

## 3. Results

**Figure 1:**
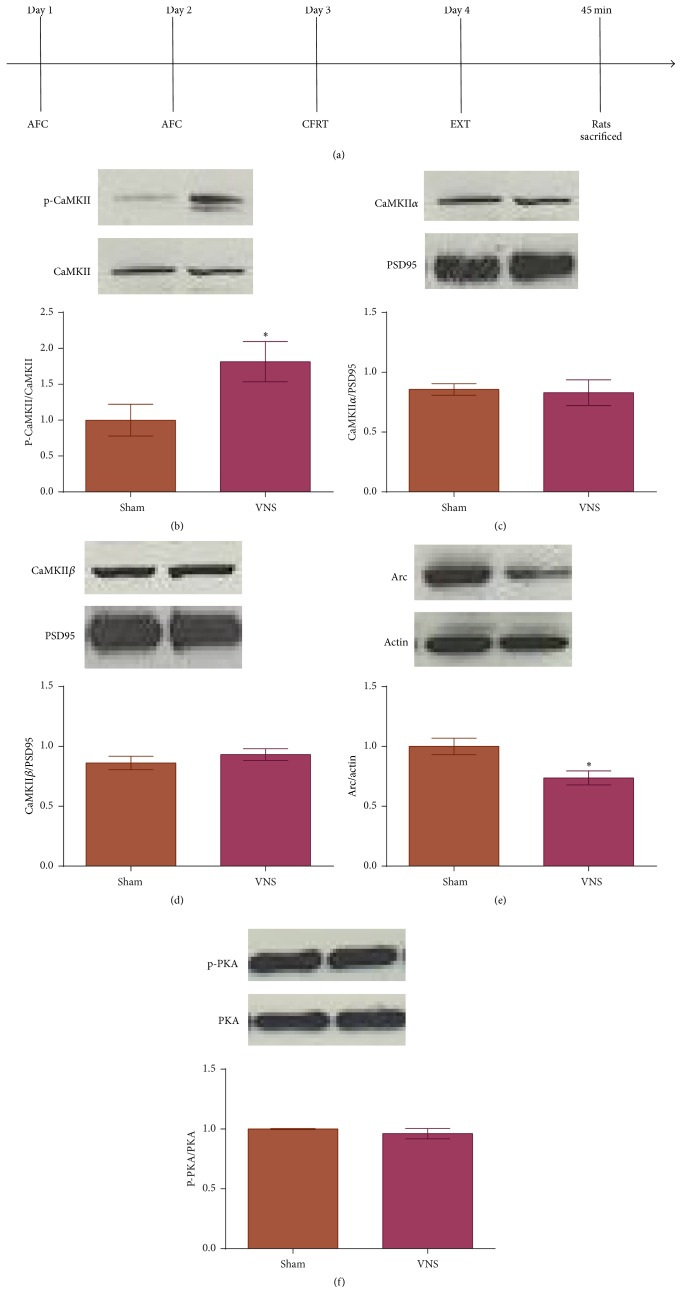
Increased expression of p-CaMKII and decreased expression of Arc protein in the BLA of animals given VNS during extinction training. (a) Timeline of behavioral protocol. On days 1 and 2 rats were subjected to auditory fear conditioning (AFC) followed by a conditioned fear response test (CFRT) on day 3. On day 4 rats underwent extinction training (EXT) and were sacrificed 45 min later. (b) Rats given VNS during extinction training show higher levels of p-CaMKII compared to rats given sham stimulation (^*∗*^ *p* < 0.05). (c, d) No significance difference in expression of CaMKII*α* or CaMKII*β* across groups. (e) VNS-treated rats show lower levels of Arc protein expression compared to sham-treated rats (^*∗*^ *p* < 0.05). (f) No difference in p-PKA expression across groups.

We assessed the state of synaptic proteins in the BLA during consolidation of fear extinction. Rats were subjected to auditory fear conditioning followed by extinction training paired with either VNS (VNS-extinction rats) or sham (sham-extinction rats) stimulation, and tissue was collected from the BLA 45 min later (Figure 1(a)). Rats given VNS during extinction training showed a significant increase in phosphorylation of CaMKII at Thr286 compared to sham-extinction rats (Figure 1(b); *t*(13) = 2.305, *p* = 0.038); no difference was seen in total levels of CaMKII*α* (Figure 1(c); *t*(6) = 0.231, *p* = 0.825) or CaMKII*β* (Figure 1(d); *t*(6) = 0.947, *p* = 0.380). Administration of VNS during extinction training also leads to a decrease in Arc protein expression in VNS-extinction rats compared to sham-extinction rats (Figure 1(e); *t*(11) = 2.907, *p* = 0.014). No difference was seen in phosphorylation of PKA at Thr197 between groups (Figure 1(f); *t*(6) = 0.881, *p* = 0.412).

**Figure 2:**
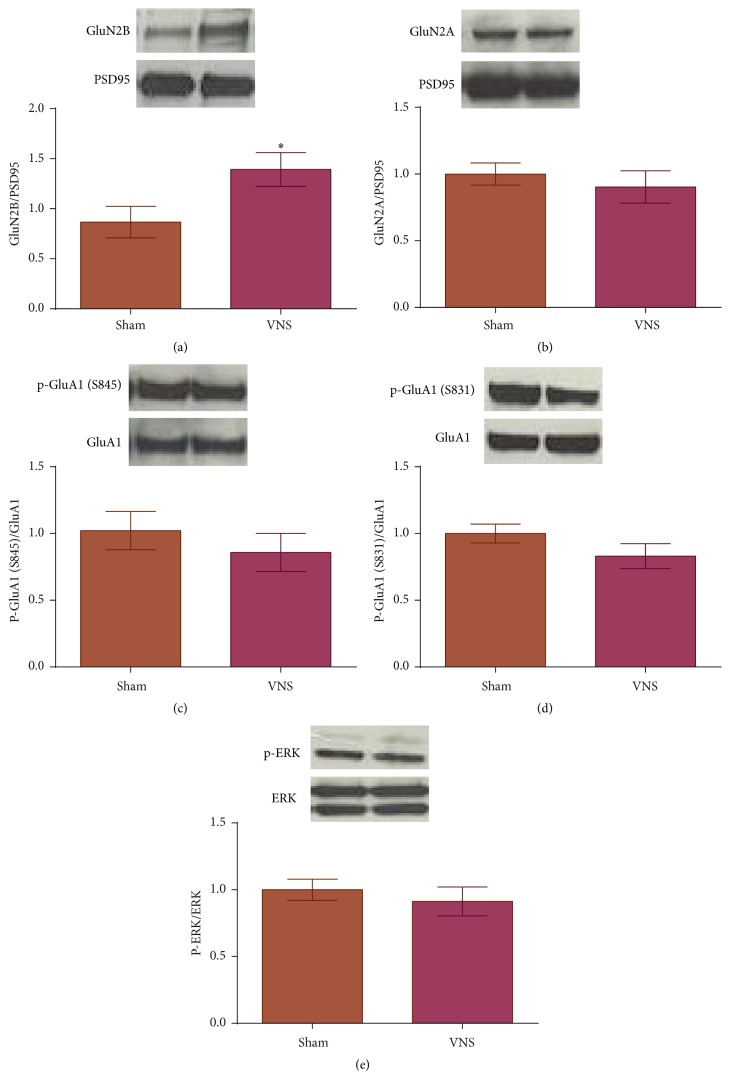
Increased expression of GluN2B in the BLA of rats given VNS during fear extinction training. (a) Rats given VNS during extinction training show higher levels of GluN2B compared to rats given sham stimulation (^*∗*^ *p* < 0.05). (b–e) No significant difference in expression of GluN2A, p-GluA1 at S845 or S831, or p-ERK across groups.

To further elucidate molecular effects of VNS-enhanced extinction, we assessed expression of downstream targets of p-CaMKII and proteins known to be affected by Arc expression that play important roles in synaptic plasticity. p-CaMKII can affect LTP through indirect phosphorylation of ERK and via interactions with both NMDA and AMPA receptors [11, 12, 14–17]. Rats given VNS during extinction training showed a significant increase in expression of the GluN2B subunit of NMDA receptors compared to sham-extinction rats (Figure 2(a); *t*(12) = 2.272, *p* = 0.042). No difference was seen in expression of the GluN2A subunit of NMDA receptors (Figure 2(b); *t*(14) = 0.662, *p* = 0.518), in phosphorylation of the GluA1 subunit of AMPA receptors at either the Ser831 (Figure 2(c); *t*(23) = 1.463, *p* = 0.157) or Ser845 site (Figure 2(d); *t*(10) = 0.811, *p* = 0.435), or in phosphorylation of ERK protein (Figure 2(e); *t*(23) = 0.659, *p* = 0.515).

**Figure 3:**
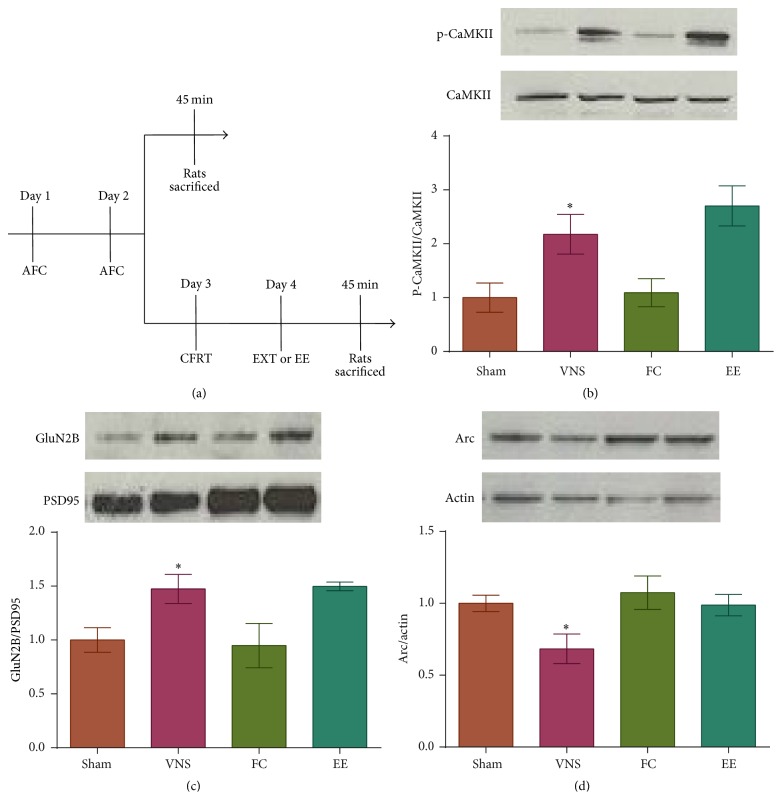
VNS accelerates molecular mechanisms of extinction learning. (a) Timeline of behavioral protocol. On days 1 and 2 rats were subjected to auditory fear conditioning (AFC). One cohort was sacrificed 45 min after AFC. Another cohort underwent a conditioned fear response test (CFRT; day 3), followed by extinction training (EXT) or extended-extinction (EE) training (day 4), and was sacrificed 45 min later. (b-c) Rats given VNS during extinction training show higher levels of p-CaMKII and GluN2B compared to rats given sham during extinction training and rats that underwent auditory fear conditioning alone (FC; ^*∗*^ *p* < 0.05) and do not differ from rats that underwent extended extinction. (d) Rats given VNS during extinction training show lower levels of Arc protein compared to all other groups (^*∗*^ *p* < 0.05).

Rats given VNS during trials of fear extinction showed significant extinction of fear, whereas rats given sham stimulation during extinction did not [7]. To determine if the molecular differences seen between VNS-treated and sham-treated rats were an effect of fear conditioning, successful extinction learning, or the treatment itself, two behavioral groups were added to the experiment: rats that underwent fear conditioning alone (fear-conditioned) and rats that reach successful extinction of fear after extended-extinction training without VNS (extended-extinction; Figure 3(a)). There was a significant difference in expression of p-CamKII at Thr286 (Figure 3(b); *F*(3,22) = 6.843, *p* = 0.002); VNS-extinction showed a significant increase in p-CaMKII compared to sham-extinction (*p* = 0.017) and fear-conditioned rats (*p* = 0.033). Expression of p-CaMKII did not differ between VNS-extinction and extended-extinction rats (*p* = 0.263). The ANOVA revealed a significant difference in expression of GluN2B (Figure 3(c); *F*(3,18) = 4.697, *p* = 0.013). GluN2B expression was significantly increased in VNS-extinction rats compared to sham-extinction (*p* = 0.018) and fear-conditioned rats (*p* = 0.013). Levels of GluN2B did not differ between VNS-extinction and extended-extinction rats (*p* = 0.907). The ANOVA revealed a significant difference in expression of Arc protein (Figure 3(d); *F*(3,22) = 3.578, *p* = 0.030); VNS-extinction rats showed a significant decrease in Arc expression compared to sham-extinction (*p* = 0.027), fear-conditioned (*p* = 0.006), and extended-extinction rats (*p* = 0.033).

**Figure 4:**
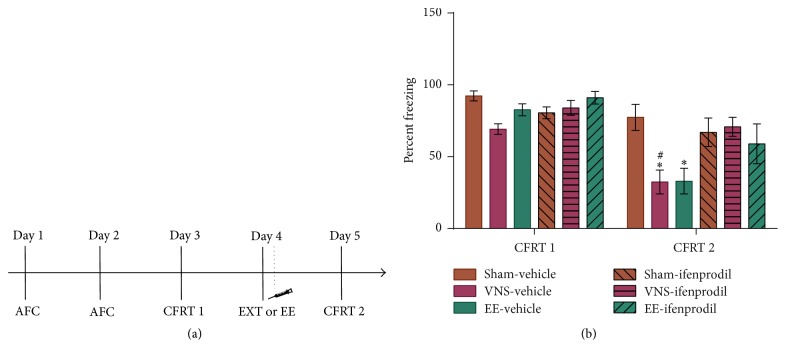
Ifenprodil blocks extinction learning in VNS-extinction and EE rats. (a) Timeline of behavioral protocol. On days 1 and 2 rats were subjected to auditory fear conditioning (AFC) followed by a conditioned fear response test (CFRT) on day 3. On day 4 rats underwent extinction training (EXT) or extended-extinction (EE) training that was immediately followed by an i.p. injection of ifenprodil or vehicle. On day 5 rats underwent a second CFRT (CFRT 2). (b) VNS-extinction and EE rats given a vehicle injection immediately after extinction training show a significant decrease in freezing levels during the second conditioned fear response test (CFRT 2) compared to sham-vehicle rats (**∗**). VNS-vehicle rats show significantly lower freezing levels during CFRT 2 compared to VNS-ifenprodil rats (#).

An increase in GluN2B expression was measured in rats that showed significant extinction of conditioned fear (VNS-extinction and extended-extinction rats; Figure 3(c)). This is consistent with previous findings indicating a critical role of GluN2B in the BLA in successful extinction of conditioned fear [21]. To determine whether GluN2B is a mediator of VNS enhancement of extinction learning, we administered the GluN2B specific antagonist ifenprodil. Rats underwent auditory fear conditioning followed by extinction training paired with VNS or sham stimulation or extended extinction. Ifenprodil or vehicle was injected (i.p.) immediately after extinction training, and conditioned fear was measured by a CFRT 24 hours later (Figure 4(a)). The ANOVA revealed a significant effect for group (Figure 4(b); *F*(5, 35) = 3.799, *p* = 0.007), with the VNS-vehicle and EE-vehicle rats showing significantly lower freezing levels during the second CFRT compared to sham-vehicle rats (versus VNS *p* = 0.006; versus EE *p* = 0.001), as well as significantly lower freezing levels in VNS-vehicle rats compared to VNS-ifenprodil rats (*p* = 0.018).

## 4. Discussion

Recent work shows that coupling VNS with exposure to the tone during fear extinction training reduces expression of the fear response faster than extinction training alone [7]. This VNS-induced enhancement in extinction learning is accompanied by changes in synaptic plasticity in the IL-BLA pathway; high frequency stimulation of the IL leads to enhanced LTP in the BLA of VNS-extinction rats but not in extended-extinction rats, whereas sham-extinction and fear-conditioned rats showed LTD in the same pathway [9]. This experiment was designed to elucidate possible mechanisms by which VNS enhances extinction of fear and affects plasticity in the IL-BLA pathway. Rats underwent auditory fear conditioning followed by extinction training paired with either VNS or sham stimulation or extended extinction. Forty-five minutes after fear conditioning or extinction training, the BLA was removed and the state of synaptic proteins was assessed. The protein kinase CaMKII plays a role in memory formation and LTP. When phosphorylated at Thr286, it forms a protein-protein complex with the GluN2B subunit of NMDA receptors which has been implicated in LTP induction and maintenance [16, 31]. Furthermore, studies suggest that expression of GluN2B in the BLA is important for fear extinction learning [21, 32]. On the other hand, Arc protein expression has been linked to fear memory formation [23, 24] and to LTD via AMPA receptor endocytosis [25, 26]. Western blot analysis was used to assess expression of CaMKII, GluN2B, and Arc in the BLA of rats after fear conditioning and after either fear extinction training paired with VNS or sham stimulation or extended extinction.

Results showed that VNS-extinction and extended-extinction rats have higher levels of p-CaMKII and the GluN2B subunit of NMDA receptors compared to fear-conditioned and sham-extinction rats. Only VNS-extinction rats showed a significant decrease in Arc protein expression compared to all other groups. Expression of GluN2B in the BLA is important for successful extinction learning and blocking GluN2B expression immediately after extinction training using the specific antagonist ifenprodil impairs consolidation of extinction of fear [33]. Because VNS-extinction rats reach similar levels of extinction and show equivalent GluN2B levels to those in extended-extinction rats, we hypothesized that VNS-paired extinction accelerates molecular mechanisms known to be important for successful extinction learning. Ifenprodil administration immediately after extinction training blocked the VNS-induced enhancement of extinction, supporting the idea that VNS works by accelerating normal mechanisms of extinction learning. Long-term potentiation is associated with an increase in CaMKII activity [34–37]. Studies dissecting the role of CaMKII in LTP have found that, once active, p-CaMKII interacts with the GluN2B subunit of NMDA receptors and the protein complex formed is important for LTP [10]. Our results suggest that LTP occurs in the BLA after extinction training, and this mechanism is accelerated in VNS-extinction rats. Furthermore, research suggests that the CaMKII/NMDAR complex plays a structural role that contributes to synaptic strengthening [38, 39]. It is possible that the increase in p-CaMKII and GluN2B results in an increase in CaMKII-NMDAR complex which in turn increases synaptic strengthening that subsequently promotes LTP in the IL-BLA pathway [9].

Arc levels were decreased in VNS-extinction rats compared to all other groups. One known role of Arc is in endocytosis of AMPA receptors [25, 26]. Arc levels are known to increase in the BLA after auditory fear conditioning [40]. Based on our results, we propose a theory that Arc expression increases in the BLA after fear conditioning leading to AMPAR endocytosis, making the pathway less excitable, increasing the disposition to LTD in the IL-BLA pathway, and predisposing synapses to LTD in the BLA of fear-conditioned and sham-extinction rats. Conversely, successful extinction learning in extended extinction leads to an increase in p-CaMKII and GluN2B, making the pathway more excitable and reversing the propensity for LTD induction in sham-extinction and fear-conditioned rats to baseline levels. VNS-paired extinction accelerates the increase in p-CaMKII and GluN2B and reduces fear conditioning-associated Arc expression, leading to NMDAR stabilization (through increased CaMKII and GluN2B), more AMPAR in the membrane (through decreased Arc-regulated AMPAR endocytosis), and, consequently, a more excitable pathway, as reported by Peña et al. [9]. Whereas our findings suggest that LTP-like mechanisms occur in the BLA during the window of extinction-memory consolidation, Cho et al. [8] analyzed synaptic changes immediately after extinction-memory recall and found a reduction of excitatory synaptic strength in the medial prefrontal cortex- (mPFC-) BLA pathway. Timing, measures, and stimulation approaches differed between the two studies. Here, we analyzed synaptic proteins during the consolidation period after extinction, and Cho et al. recorded BLA responses following optogenetic stimulation of mPFC axons in the BLA after extinction recall. The molecular results presented here reflect protein expression in the BLA and are not specific to the IL-BLA pathway.

In the postsynaptic density, CaMKII can also modulate activity of AMPA receptors [41, 42]; activation of CaMKII leads to phosphorylation of the GluA1 subunit of AMPA receptors at the Ser831 site, a process that promotes LTP by increasing AMPA receptor conductance [13, 43]. Research shows that phosphorylation of GluA1 at Ser831 correlates with LTP, whereas phosphorylation of GluA1 at Ser845 is associated with trafficking to extrasynaptic regions and LTD [13]. In our studies we saw no significant difference in phosphorylation of GluA1 across groups. Another research shows that inactivation of AMPA receptors in the BLA during extinction of fear blocks the initial freezing response to the conditioned stimulus but does not affect long-term memory of extinction of fear. Conversely, blocking NMDA receptors in the BLA does not affect initial freezing to the conditioned stimulus but blocks long-term memory of extinction [44], suggesting that AMPA receptors in the BLA are involved in* expression *of conditioned fear whereas NMDA receptors in the BLA play a role in* extinction *of conditioned fear. In line with this study, our results suggest that VNS-paired extinction might work through an NMDA rather than AMPA-dependent mechanism to enhance extinction of fear. D-cycloserine is a partial agonist of NMDA receptors, and administration of d-cycloserine before extinction training enhances consolidation of extinction of fear [45]. DCS has shown promising results in the clinical field as an adjunct to exposure therapy [46, 47], a form of cognitive behavioral therapy (CBT) that is frequently used with the goal of extinguishing conditioned fears. Like DCS, VNS might work through NMDA receptors to enhance extinction of conditioned fear. Although direct stimulation of the vagus nerve requires a surgery, the spatial and temporal control of peripheral nerve stimulation exceeds that with drug administration, and noninvasive transcutaneous VNS is currently under investigation in humans for optimization as an adjunct to exposure therapy [48].

Metaplasticity was defined by Abraham and Bear [49] as the plasticity of synaptic plasticity. Metaplasticity refers to synaptic activation or synaptic change that can influence subsequent induction of synaptic plasticity. The mechanisms of metaplasticity overlap greatly with the mechanisms of synaptic plasticity, with evidence suggesting a role for NMDA receptor activation, rises in postsynaptic calcium, and subsequent regulation of downstream components like protein kinases and phosphatases [49–54]. In studies of molecular correlates of LTP and LTD, it is important to consider that the changes observed can just as likely relate to metaplasticity as to synaptic plasticity. The studies reported by Peña et al. [9] provide evidence that VNS treatment during extinction training produces metaplasticity in the IL-BLA pathway. The Peña et al. [9] study shows that VNS-enhanced extinction predisposes the synapses towards potentiation, resulting in LTP when the IL-BLA pathway was stimulated 48 hours after VNS-paired extinction training. The initial synaptic changes that occur after VNS-paired extinction training and modulate subsequent plasticity are unknown. The results reported here suggest that increased phosphorylation of CaMKII, increased levels of GluN2B, and decreased Arc protein expression are some of the metaplastic changes that facilitate LTP in the IL-BLA pathway.

VNS-enhanced extinction leads to increased expression of synaptic proteins associated with LTP and decreased expression of Arc protein, which is associated with AMPA receptor endocytosis and LTD [25, 26]. Expression of GluN2B is increased in the BLA of VNS-treated rats and rats given extensive extinction training and blockade of GluN2B impairs extinction of conditioned fear, indicating that the upregulation of GluN2B expression plays a necessary role in the VNS-induced enhancement of extinction. An increase in p-CaMKII may promote LTP by forming a complex with GluN2B and enhancing NMDA receptor function. These results are consistent with previous findings indicating that VNS enhances extinction of conditioned fear in rats and promotes plasticity in the IL-BLA pathway by predisposing synapses to induction of LTP.
